# Editorial of Special Issue “Latest Multifactorial Developments on Neuropsychiatric Disorders and Manifestations”

**DOI:** 10.3390/cimb47030183

**Published:** 2025-03-12

**Authors:** Cătălina Ionescu, Alin Ciobica

**Affiliations:** 1 Department of Biology, Faculty of Biology, Alexandru Ioan Cuza University of Iasi, Bd. Carol I No. 20A, 700505 Iasi, Romania; catalinaionescu81@yahoo.com; 2 “Ioan Haulica” Institute, Apollonia University, Păcurari Street 11, 700511 Iasi, Romania; 3 Center of Biomedical Research, Romanian Academy, Iasi Branch, Teodor Codrescu 2, 700481 Iasi, Romania; 4 Academy of Romanian Scientists, Str. Splaiul Independentei No. 54, Sector 5, 050094 Bucharest, Romania; 5 CENEMED Platform for Interdisciplinary Research, University of Medicine and Pharmacy “Grigore T. Popa”, 700115 Iasi, Romania

This Special Issue, entitled “Latest Multifactorial Developments on Neuropsychiatric Disorders and Manifestations”, underscores the multifactorial nature of neuropsychiatric disorders, highlighting the complex interplay between genetic, molecular, and environmental factors. Here, the studies included have provided significant contributions to our understanding of the biological underpinnings of disorders such as Alzheimer’s disease, Parkinson’s disease, schizophrenia, autism, anxiety, and depression for various genetic factors, offering valuable information that has been of interest in further studies [1,2,3,4,5,6].

One of the key findings discussed is the upregulation of complement component C3 in the hippocampus following lipopolysaccharide stimulation in a fragile X syndrome model, shedding light on neuroinflammatory processes that may exacerbate cognitive dysfunction in neurodevelopmental disorders. This supports the growing recognition of immune system involvement in neuropsychiatric conditions and its potential as a therapeutic target [7].

Further advancing our knowledge of Parkinson’s disease, research on mitochondrial dysfunction in dopaminergic neurons derived from patients with LRRK2- and SNCA-associated genetic variants has reinforced the critical role of bioenergetic deficits in neurodegeneration. These findings open new avenues for therapeutic strategies aimed at restoring mitochondrial function, which could be pivotal in slowing disease progression [8].

Another compelling study focused on the genetic basis of autism spectrum disorder (ASD), particularly the association of OXTR, AVPR1a, LNPEP, and CD38 gene expression with ASD clinical presentation. The findings support the notion that neurodevelopmental disorders have a strong genetic component that modulates social behavior and cognitive functioning. Understanding these genetic influences could pave the way for more targeted interventions in ASD [9].

Furthermore, this Issue examines the structural variations of prions and prion-like proteins associated with neurodegeneration, reinforcing the idea that protein misfolding and aggregation are fundamental pathological mechanisms in several neurodegenerative diseases. Given the role of these proteins in conditions such as Alzheimer’s and Parkinson’s, future research should further explore their implications in disease onset and progression, as well as their potential as biomarkers or therapeutic targets [10].

Finally, the identification of a novel mutation in the Sacsin gene (p.Val1335IIe) as a possible cause of late-onset sacsinopathy suggests that genetic haploinsufficiency may contribute to certain neurodegenerative conditions. This finding highlights the importance of genetic screening and personalized medicine approaches in understanding and managing late-onset neurological disorders [7].

Together, these studies reinforce the need for an integrated, multidisciplinary approach to neuropsychiatric disorders, moving beyond reductionist models to embrace their multifactorial nature. In fact, almost any unidirectional theory regarding the pathophysiology of neuropsychiatric disorders has, at present, been widely abandoned.

Thus, future research should continue to explore biomarkers for early diagnosis and therapeutic interventions, in addition to the intricate genetic and molecular interactions that drive disease pathology. Advancing our knowledge in these areas will be crucial for developing more precise, personalized, and effective treatments for these disorders.

In this way, by fostering collaboration across disciplines, we can move closer to a comprehensive understanding of neuropsychiatric diseases and ultimately improve patient outcomes.
